# Resting energy expenditure and carbohydrate oxidation are higher in elderly patients with COPD: a case control study

**DOI:** 10.1186/1475-2891-11-37

**Published:** 2012-06-06

**Authors:** Bruna Rubi Ramires, Erick Prado de Oliveira, Gustavo Duarte Pimentel, Kátia Cristina Portero McLellan, Darlan Muller Nakato, Márcia Maria Faganello, Maurício Longo Galhardo, Luciene de Souza Venâncio

**Affiliations:** 1 Universidade Metodista de Piracicaba (UNIMEP), Lins, SP, Brazil; 2 Department of Pathology, São Paulo State University (UNESP), Botucatu, SP, Brazil; 3 Department of Public Health, Centre for Physical and Nutritional Metabolism (CeMENutri), São Paulo State University (UNESP), Botucatu, SP, Brazil; 4 Department of Internal Medicine, Campinas State University (UNICAMP), Campinas, SP, Brazil; 5 Department of Physiotherapy, Centro Universitário Católico Salesiano Auxilium-Unisalesiano, Lins, SP, Brazil; 6 CeMENutri - Faculdade de Medicina, Depto. de Saúde Pública (FMB - UNESP), Distrito de Rubião Jr, s/n°, 18.618-970, Botucatu, SP, Brazil

**Keywords:** Chronic obstructive pulmonary disease, Resting energy expenditure, Elderly, Carbohydrate oxidation

## Abstract

**Background:**

Elderly patients with chronic obstructive pulmonary disease (COPD) usually have a compromised nutritional status which is an independent predictor of morbidity and mortality. To know the Resting Energy Expenditure (REE) and the substrate oxidation measurement is essential to prevent these complications. This study aimed to compare the REE, respiratory quotient (RQ) and body composition between patients with and without COPD.

**Methods:**

This case–control study assessed 20 patients with chronic obstructive pulmonary disease attending a pulmonary rehabilitation program. The group of subjects without COPD (control group) consisted of 20 elderly patients attending a university gym, patients of a private service and a public healthy care. Consumption of oxygen (O_2_) and carbon dioxide (CO_2_) was determined by indirect calorimetry and used for calculating the resting energy expenditure and respiratory quotient. Body mass index (BMI) and waist circumference (WC) were also measured. Percentage of body fat (%BF), lean mass (kg) and muscle mass (kg) were determined by bioimpedance. The fat free mass index (FFMI) and muscle mass index (MMI) were then calculated.

**Results:**

The COPD group had lower BMI than control (p = 0.02). However, WC, % BF, FFMI and MM-I did not differ between the groups. The COPD group had greater RQ (p = 0.01), REE (p = 0.009) and carbohydrate oxidation (p = 0.002).

**Conclusions:**

Elderly patients with COPD had higher REE, RQ and carbohydrate oxidation than controls.

## Introduction

Chronic obstructive pulmonary disease (COPD) is characterized by an obstructed or chronically limited airflow which progresses slowly and irreversibly. The main cause of COPD is the combination of chronic bronchitis and pulmonary emphysema, due mainly to smoking [1].

Patients with COPD usually have a compromised nutritional status which is an independent predictor of morbidity and mortality [2,3]. Malnutrition affects approximately one-third of the adult patients with this disease [2], mainly in elderly [4]. The fat-free mass decreases and body fat increases but body mass index (BMI) remains unchanged, suggesting that one body compartment is replaced by another [5]. Malnutrition in patients with COPD is associated with increased energy expenditure due to hypermetabolism, which in turn is caused by greater respiratory muscle effort, oxygen requirement and inflammation and lower food intake [6].

It is essential to determine resting energy expenditure (REE) to prevent a negative caloric balance and consequently malnutrition, since REE is the main component of total energy expenditure (TEE) [7] and is vital for adequate food intake [8]. Additionally, it is also critical to know which energy substrate is the most oxidized so that appropriate dietary adjustments can be made.

Changes in energy metabolism stemming from patients with COPD can be detected by indirect calorimetry, which measures oxygen consumption (O_2_) and elimination of carbon dioxide (CO_2_), thus determining REE and respiratory quotient (RQ). RQ depends on the mixture of energy substrates (carbohydrates, fats and proteins) that is the mixture metabolized during rest or exercise. In patients with COPD, a RQ < 1.0 is desirable since the patient will be exhaling less carbon dioxide [9]. However, no study assessed carbohydrate and fat oxidation separately in this group of patients (with COPD), and this is essential for understanding the energy metabolism of these individuals. An appropriate nutritional strategy for treating malnutrition in patients with COPD has not yet been established because, despite extensive studies on their energy metabolism, not much data is available on their oxidative capacity [10].

Hence, the aim of the present study was to compare the oxidation amount of energy substrates and REE of elderly patients with and without COPD.

## Methodology

### Sample

The study was conducted from December 2008 to February 2009. Forty elderly individuals living in the same area were recruited and divided into two groups: a COPD group (16 males and 4 females) and a control group (16 males and 4 females). Patients in the COPD group were diagnosed according to the Global Initiative for Chronic Obstructive Lung Disease – (Gold) [11] criteria and were attending a pulmonary rehabilitation program provided by a private healthcare service. The controls and COPD patients were paired by gender and age.

The study was approved by the Methodist University of Piracicaba Research Ethics Committee under protocol number 40/09. All study participants signed a free and informed consent form.

### Study design

A case–control study was done to compare the body composition, REE and RQ of individuals with and without COPD.

The inclusion criteria for the COPD patients were: age ≥ 60 years and the presence of pulmonary emphysema and/or chronic bronchitis. The inclusion criteria for the control group were: age ≥ 60 years and not having pulmonary diseases.

### Assessment of body composition

Weight (kg) and height (cm) were measured for determining body mass index (BMI). Nutritional status was classified according to the World Health Organization (WHO) [12]. Waist circumference was measured midway between the lowest rib and the iliac crest with an inelastic tape measure and classified according to the Expert Panel on Detection, Evaluation and Treatment of High Blood Cholesterol in Adults (NCEP-ATPIII) [13]. Bioelectrical impedance (BioDynamics model 310) was used for determining fat and muscle masses. Absolute fat and fat-free mass (FFM) were calculated by the Segal et al. (1988) equations [14]. Muscle mass (MM) was calculated by the Janssen et al. (2000) equation [15]. Then, the muscle mass index (MM-I) was calculated by dividing MM by the square of the height and were classified the sarcopenia [16].

### Assessment of REE and substrate oxidation

Indirect calorimetry (Metalyzer 3B – R2 (Cortex), breath by breath) was used for determining REE, RQ and carbohydrate and fat oxidation after calibration of barometric pressure (960 mbar) and room air (O_2_: 20.93/CO_2_: 0.03 Vol%) against a known gas mixture (White Martins, O_2_: 15.94/CO_2_: 5.01 Vol%) and volume (3-liter Hans Hudolph syringe, V = 3.0 L). REE was determined by the Weir (1949) [17] equation after measuring oxygen consumption (VO_2_) and production of carbon dioxide (VCO_2_). RQ was determined by the respiratory exchange ratio (VCO_2_/VO_2_). The Jeukendrup & Wallis (2005) [18] equation was used for determining carbohydrate and fat oxidation separately.

For the measurements, the fasting participants were asked to lie on their backs using a mask and remain still and silent until the steady state condition was reached over a 5-minute period and 20% variation of VO_2_, 12% variation of VCO_2_ and 10% variation of RQ. Both room temperature (25.0°C ± 2.0) and relative humidity (47.0 ± 4.5%) were controlled.

None of the individuals presented the flu, cold or cough during the assessment and all individuals were asked to avoid exercise and caffeinated or alcoholic beverages consumption during the 48 hours that preceded the assessment.

### Statistical analysis

The results were expressed as mean ± standard deviation and percentage. The software Statistica 6.0 was used for the statistical analyses. The qualitative variables (age, gender, race/color, marital status, occupation, education level, place of residence, nutritional status, smoking status, high blood pressure, presence of diabetes mellitus type 2 and level of physical activity) were compared by the chi-square or Fisher’s exact test. The Shapiro-Wilk test was used for testing for normality. Since all variables were normal, the unpaired Student’s *t*-test was used for comparing the quantitative variables of both groups. The significance level was set at 5%.

## Results

### Sociodemographic and clinical assessment

The study sample consisted of elderly, white, non-working individuals, mostly males. The COPD group had higher education level and higher percentage of smokers and physically active individuals than the control group (Table 1).

**Table 1:** Sociodemographic and clinical characteristics of the chronic obstructive pulmonary disease and control groups

**Characteristics**	**Control group**		**COPD group**		**p**
	**n = 20**		**n = 20**		
	**n**	**%**	**n**	**%**	
**Gender**					1.000
Males	16	80	16	80	
Females	4	20	4	20	
**Age**					0.47
(years)	75.5± 7.65 (61-86)		73.7± 8.0 (61-86)		
**Race/Color**					1.0000
White	17	85	17	85	
Non-white	3	15	3	15	
**Marital status**					0.1504
Married	16	80	13	65	
Widowed	1	5	5	25	
Divorced	1	1	2	10	
Single	2	10	-	-	
**Occupation**					0.5483
Inactive	19	95	18	90	
Active	1	5	2	10	
**Education level**					0.0274*
Illiterate	-	-	3	15	
Elementary school	9	45	11	55	
High school	8	40	1	5	
Higher education	3	15	5	25	
**Place of residence**					0.03501*
Lins	20	100	16	80	
Other cities	-	-	4	20	
**Nutritional status**					0.0705
Normal weight	11	55	12	60	
Underweight	-	-	3	15	
Overweight	5	25	5	25	
Obese	4	20	-	-	
**Duration of COPD**	-	-	4.65 ± 2.97	-	-
**Smoking status**					0.00091*
Non-smoker	12	60	2	10	
Ex-smoker and smoker	8	40	18	90	
**High blood pressure**	9	45	9	45	1.000
**Diabetes mellitus type 2**	-	-	3	15	0.0717
**Physical activity**					0.03501*
Active	16	80	20	100	
Inactive	4	20	-	-	

COPD: Chronic obstructive pulmonary disease; ^*^ = p indicates a significant difference between the groups; Gender, race/color, marital status, occupation, education level, nutritional status: chi-square test; place of residence: Fisher’s exact test; age: unpaired Student’s *t*-test.

### Assessment of body composition

Nutritional status, classified according to BMI, did not differ significantly between the groups (Table 2) but the COPD group had lower BMI than the control group. MM-I, degree of sarcopenia (%), body fat (%), fat-free mass index (FFM-I) and waist circumference (cm) did not differ between the groups, showing that both groups had similar body composition (Table 2).

**Table 2:** Body composition of the chronic obstructive pulmonary disease (COPD) and control groups

	**Control**	**COPD**	***p***
**Age (years)**	75.5 ± 7.7	73.7 ± 8.0	0.47
**Weight (kg)**	68.1 ± 12.4	61.0 *+* 12.1	0.054
**Height (cm)**	162.8 ± 6.5	163.7 ± 7.9	0.70
**BMI (kg/m**^**2**^**)**	25.6 ± 4.1	22.8 ± 3.4	0.02
**WC (cm)**	94 ± 10.9	89.7 ± 14.2	0.28
**% body fat**	28.9 ± 3.4	26.9 ± 3.3	0.07
**Muscle mass (kg)**	22.3 ± 5	21.4 ± 5	0.57
**MM-I (kg/m**^**2**^**)**	8.4 ± 1.5	7.9 ± 1.5	0.32

BMI: body mass index; WC: waist circumference; MM-I: muscle mass index.

### Assessment of REE and substrate oxidation

**Figure 1:**
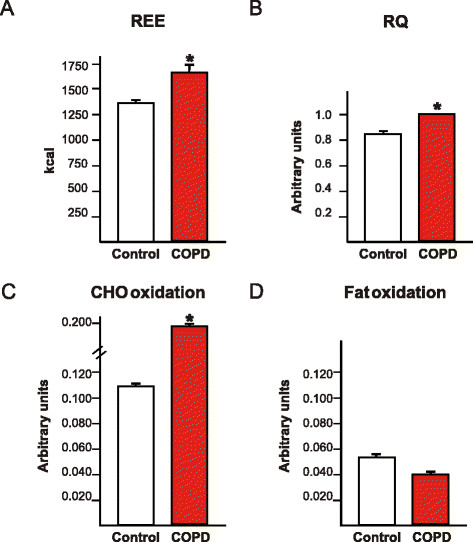
Assessment of resting energy expenditure, respiratory quotient and carbohydrate and fat oxidation of chronic obstructive pulmonary disease patients and controls.

Individuals with COPD had higher REE, carbohydrate oxidation and RQ than the controls. Fat oxidation did not differ between the groups (Figure 1).

## Discussion

In the present study, we found that individuals with COPD had higher REE and resting carbohydrate oxidation than the controls, regardless of body composition, since both groups had similar muscle mass.

Other studies have also found that patients with COPD have higher REE (approximately 15 to 26%) [6,19]. The greater energy expenditure of individuals with COPD is probably due to increased respiratory muscle effort and inflammatory mediators, in addition to the effects of medication (oral or systemic corticosteroids, theophylline, hormones, benzodiazepines and antipsychotics) [19-21].

This study found that mean RQ and carbohydrate oxidation were higher in the COPD group, while fat oxidation was similar in both groups. Increased carbohydrate oxidation in the COPD group was probably caused by increased anaerobic metabolism due to reduced ability to capture oxygen [22]. When carbohydrates are oxidized in the absence of oxygen, only 2 ATP molecules per millimol of carbohydrates are generated, while the presence of oxygen increases ATP/millimol generation to 36 ATPs [22]. Hence, individuals with COPD need to oxidize greater amounts of carbohydrates than healthy individuals to generate similar amounts of ATP molecules, because COPD patients present a higher ATP cost [23].

Moreover, individuals with COPD show depletion of type I muscle fibers (aerobic) and a greater proportion of type IIx fibers, with consequent reduction in the number of mitochondria and oxidative enzymes and lower expression of peroxisome proliferator-activated receptors (PPAR-α) and PGC-1α, molecular markers that are related to mitochondrial biogenesis and higher activation of oxidative metabolism [24]. Although individuals with COPD have lower aerobic metabolism, fats are only oxidized in the presence of oxygen [9], however their fat oxidation was not affected, which is necessary more studies to explain this fact.

BMI has been used as a good indicator of the nutritional status of individuals with COPD [25]. However, it is not possible to determine body composition with this index [26]. So this study also determined the body composition, muscle mass index, degree of sarcopenia, percentage of body fat, fat-free mass index and waist circumference of the participants.

The results showed that the COPD and control groups had similar body composition, which may be explained by the advanced age of the members of both groups [27]. It is estimated that people lose 5% of their muscle mass per decade after age 40 years and this loss intensifies after age 65 years [27]. The degree of sarcopenia was similar in both groups since both groups presented loss of muscle mass. However, loss was greater in individuals with COPD and class II sarcopenia than in controls. A similar result was found by Sergi et al., 2006, [19] who found a greater prevalence of sarcopenia in individuals with COPD than in healthy controls. COPD can intensify the loss of muscle mass, affecting respiratory and peripheral muscle performance, and thereby reducing ventilation and physical activity [28]. According to the II Brazilian Consensus on COPD [1], patients with moderate COPD may have FFM depletion involving skeletal muscles, which results in weight loss. In the present study, the FFM-I of both groups was similar. Sergi et al.*,* 2006, [19] found that individuals with COPD had lower FFM than healthy controls. Reduced FFM in these patients could be associated with age and particularly regard peripheral skeletal muscles, which may manifest as weakness in individuals with COPD [19].

The present and other Brazilian studies have found that COPD affects mainly elderly males with a past or current history of smoking [1,29]. Another aspect observed in individuals with COPD is the change in nutritional status, such as malnutrition [30]. However, a recent study [19] found a higher prevalence of normal weight individuals. It is important for individuals with COPD not to become underweight since the severity of pulmonary diseases is associated with low BMI, which can result in worse nutritional prognosis and shorter survival [2]. The COPD participants of the present study were attending a pulmonary rehabilitation program and mean duration of disease was 4.65 years, that is, they were in the initial phase of the disease. Hence, this may explain the high prevalence of normal weight or overweight individuals in the present study.

Although most patients were normal weight or overweight, the risk of malnutrition was found in 15% of the patients with COPD. This risk was not observed in the control group. Progressive weight loss in these patients may be due to a set of factors, such as release of inflammatory mediators, which can contribute to hypermetabolism and generate an inappropriate response to food intake, and reduced food intake [31]. Moreover, increased serum concentration of leptin has already been observed and now it is known that this hormone promotes greater energy expenditure and consequently, greater weight loss [31]. Recently, Ying et al. (2008) [32] found that underweight individuals with COPD have high serum levels of ghrelin. Ghrelin reduces fat oxidation, which may explain why the COPD participants of the present study did not present more fat oxidation than the controls. However, more studies are necessary to explain these findings.

Diet affects nutritional status and may change the progression rate of pulmonary disease [33]. Recent studies do not recommend high-fat diets, but an energy intake that allows individuals to reach or maintain an appropriate nutritional status [3]. A high-fat, low-carbohydrate diet can improve the prognosis of COPD patients with acute exacerbation [34]. Patients with marginal ventilatory reserve might benefit from a high-fat diet [35]. Although the present study did not assess the pulmonary ventilation of the participants, it has found that carbohydrates are the main substrates oxidized. The recommended amount of carbohydrates is 50 to 60% of the total energy expenditure of the patient. Excessive carbohydrate intake could cause lipogenesis with consequent excessive production of carbon dioxide and hepatic steatosis, which in turn increases RQ and production of carbon dioxide, resulting in respiratory failure [36]. Hence, the recommended carbohydrate intake of 50 to 60% of the total energy expenditure seems not to induce hyperventilation and also avoids malnutrition by replacing the burned carbohydrates.

In the group studied in this work it is possible to conclude that elderly patients with COPD have higher REE, RQ and carbohydrate oxidation than healthy controls.

## Abbreviations

COPD: Chronic obstructive pulmonary disease; REE: Resting Energy Expenditure; BMI: Body Mass Index; RQ: Respiratory Quotient; NCEP-ATPIII: Expert Panel on Detection; Evaluation and Treatment of High Blood Cholesterol in Adults; FFM: Fat-free mass; MM: Muscle mass; MM-I: Muscle mass index; WC: Waist Circumference.

## Competing interests

The authors declare that they have no competing interests.

## Authors’ contributions

BRR, DMN, MLG and MMF collected the data and elaborated the manuscript. EPO, GDP, LSV wrote and corrected the manuscript. KCPM revised the final manuscript. All authors read and approved.
